# The Electronic Structures and Optical Properties of Alkaline-Earth Metals Doped Anatase TiO_2_: A Comparative Study of Screened Hybrid Functional and Generalized Gradient Approximation

**DOI:** 10.3390/ma8085257

**Published:** 2015-08-24

**Authors:** Jin-Gang Ma, Cai-Rong Zhang, Ji-Jun Gong, You-Zhi Wu, Sheng-Zhong Kou, Hua Yang, Yu-Hong Chen, Zi-Jiang Liu, Hong-Shan Chen

**Affiliations:** 1 School of Sciences, Lanzhou University of Technology, Lanzhou 730050, China; E-Mails: decetwen1989@163.com (J.-G.M.); gongjijun@163.com (J.-J.G.); hyang@lut.cn (H.Y.); chenyh@lut.cn (Y.-H.C.); 2 State Key Laboratory of Advanced Processing and Recycling of Non-ferrous Metals, Lanzhou University of Technology, Lanzhou 730050, China; E-Mails: youzhiwu@163.com (Y.-Z.W.); kousz@lut.cn (S.-Z.K.); 3 Department of Physics, Lanzhou City University, Lanzhou 730070, China; E-Mail: liuzj_scu@126.com; 4 College of Physics and Electronic Engineering, Northwest Normal University, Lanzhou 730070, China; E-Mail: chenhs@nwnu.edu.cn

**Keywords:** alkaline-earth metal, anatase TiO_2_, doping mechanism, electronic structures, density functional theory

## Abstract

Alkaline-earth metallic dopant can improve the performance of anatase TiO_2_ in photocatalysis and solar cells. Aiming to understand doping mechanisms, the dopant formation energies, electronic structures, and optical properties for Be, Mg, Ca, Sr, and Ba doped anatase TiO_2_ are investigated by using density functional theory calculations with the HSE06 and PBE functionals. By combining our results with those of previous studies, the HSE06 functional provides a better description of electronic structures. The calculated formation energies indicate that the substitution of a lattice Ti with an AEM atom is energetically favorable under O-rich growth conditions. The electronic structures suggest that, AEM dopants shift the valence bands (VBs) to higher energy, and the dopant-state energies for the cases of Ca, Sr, and Ba are quite higher than Fermi levels, while the Be and Mg dopants result into the spin polarized gap states near the top of VBs. The components of VBs and dopant-states support that the AEM dopants are active in inter-band transitions with lower energy excitations. As to optical properties, Ca/Sr/Ba are more effective than Be/Mg to enhance absorbance in visible region, but the Be/Mg are superior to Ca/Sr/Ba for the absorbance improvement in near-IR region.

## 1. Introduction

Titanium dioxide (TiO_2_) has been widely applied in the systems of pigment, photocatalysis, hydrogen storage and production, novel solar cells, and so on [1,2,3,4,5,6,7,8,9,10], because TiO_2_ has many merits, including nontoxicity, high stability, abundant resource, *etc.* [11,12]. However, the wide intrinsic band gaps of TiO_2_ (3.2 eV for anatase and 3.0 eV for rutile) crucially limit the practical applications involving solar energy, such as photocatalyst, dye sensitized solar cells (DSSCs), perovskite solar cells, and other devices/equipments. To enhance the utilization efficiency of solar energy, the requirement is the reduction of TiO_2_ band gaps, so that the absorption properties might be matching well with solar spectra. Among the reported natural polymorphs of TiO_2_ (rutile, anatase, and brookite) [13], anatase phase commonly exists in TiO_2_ nano-scale materials [9]. Therefore, the modification of electronic structures and related properties for anatase TiO_2_ is very important for the applications of TiO_2_ nano-materials.

Doping is the convenient method to tailor material properties. The electronic structures of TiO_2_ can be well tuned by doping due to its good ability of solvent for numerous impurities [14]. As a photocatalysis material, it had been reported that the photocatalytic properties of TiO_2_ were enhanced by alkaline-earth metallic dopant, such as Be [15,16], Ca [17], and Sr [18]. Meanwhile, the increasing of open-circuit voltage (*V*_oc_) was reported for DSSC fabricated by using Mg-doped anatase-TiO_2_ electrode [19]. The outperformance of perovskite solar cells with thin dense Mg-doped TiO_2_ as hole-blocking layers was also reported [20,21]. Therefore, alkaline-earth metallic (AEM) dopant can improve the performance of TiO_2_ in photocatalysis and solar cells. However, even only for Mg-doped TiO_2_, the doping effects on electronic structures have not been well recognized from experiment. For instance, the analysis of the *V*_oc_ of DSSC under different surface charge densities deduced that the Mg-doped anatase TiO_2_ samples induced the negative shift of the conduction bands (CBs) [22]. Whereas, the Mott-Schottky plot suggested that the Mg-doped anatase TiO_2_ photoanode shifted the flat band potential positively [23]. The improved performance of perovskite solar cells with Mg-doped anatase TiO_2_ was attributed to the better properties of Mg-modulated TiO_2_ as compared to TiO_2_, such as upshifted CB minimum and downshifted valence band (VB) maximum, *etc.* [20]. Therefore, it is necessary to study on the doping mechanism of AEM-doped anatase TiO_2_.

On the other hand, electronic structure calculations are effective method to investigate the doping mechanism and to understand the related properties [24]. For example, Nguyen and co-workers examined the influences of metallic X dopants (X = Be, Mg, Ca, Zn, Al, W and Nb) on the electronic structures of anatase TiO_2_ based upon density functional theory (DFT) calculations [25]. Based upon GGA + *U* calculations (*U* = 4.2 eV for Ti), it predicted that a small-polaronic Ti^3+^ gap state existed within the semiconducting system for Nb, Ta-doped rutile and anatase TiO_2_ [26]. In terms of LDA + *U* calculations (the *U* values of 7.51 and 4.37 eV for Ti and O, respectively), the Mg dopant was able to enhance the optical absorption efficiency for anatase TiO_2_, especially in the near-infrared region [27]. However, the proper *U* value depends upon the investigated properties [28]. Therefore, the different values of correction parameter *U* for on-site Coulomb interactions curiously reduce the comparability of computational studies.

The local or semi-local approximations of traditional DFT usually lead to erroneous descriptions of material properties related electronic structures, such as band gap, *etc*. One way to remedy this deficiency is to use hybrid functional, where the portion of the nonlocal Hartree-Fock type exchange is admixed with a semi-local exchange-correlation functional. Thanks to Heyd, Scuseria, and Ernzerhof, they developed the range separated hybrid functional based on a screened Coulomb potential for the exchange interaction, commonly known as HSE functional [29,30]. It has been found that HSE functional was superior to other functionals for the description electronic structures of strong correlated systems, such as Fe-based superconductors [31] and point defects in TiO_2_ [32].

In this work, in order to understand the doping mechanism, the formation energies, electronic structures and optical properties of anatase TiO_2_ doped by AEM (Be, Mg, Ca, Sr, Ba) are investigated by using DFT calculations. Furthermore, the screened hybrid density functional and generalized gradient approximations (GGA) are applied in order to address the functional effects in DFT. The doping mechanisms are analyzed based upon the calculated results. The results agree with those of the available experiments and other theoretical works.

## 2. Results and Discussion

### 2.1. Local Structures 

**Figure 1:**
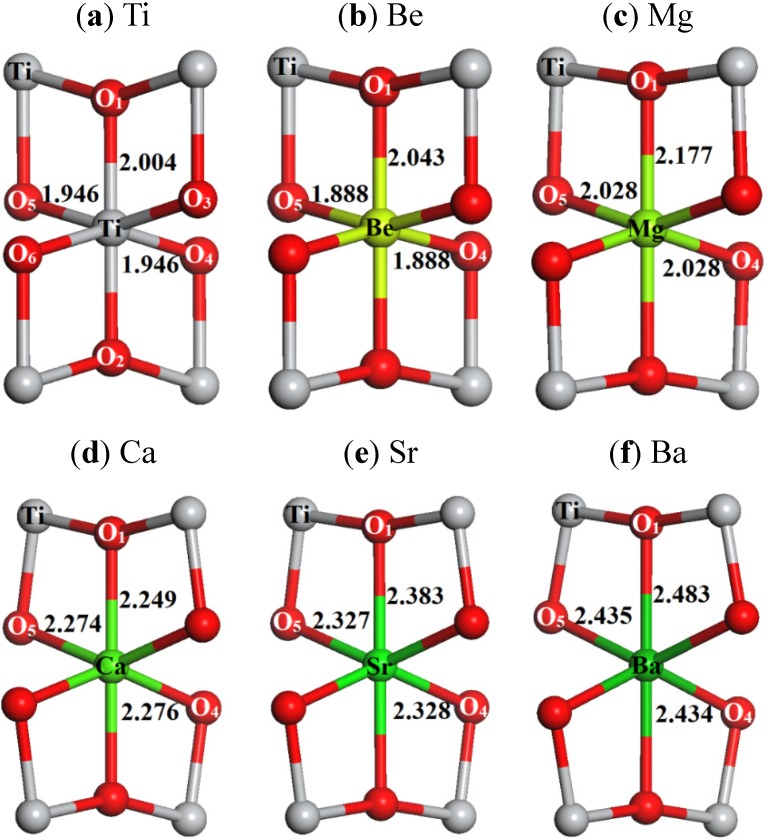
The relaxed local structures of AEM-doped anatase TiO_2_, calculated by using the PBE functional: (**a**) undoped TiO_2_; (**b**) Be-doped; (**c**) Mg-doped; (**d**) Ca-doped; (**e**) Sr-doped; (**f**) Ba-doped. The bond lengths are given in angstroms.

The substitution effects of AEM dopant on structures can be seen from the local geometries around AEM atom. Table 1 summarizes the bond lengths in the local AEMO_6_ octahedron (see Figure 1). It can be seen that the local structure deformations exist in all cases due to introducing of dopant. For instance, in the case of Mg-doped TiO_2_ (see Figure 1c), the calculated bond lengths of two different Mg-O bonds are about 0.082 and 0.173 Å longer than those of two corresponding Ti-O bonds in undoped TiO_2_, which are 1.946 and 2.004 Å, respectively. Also, the bond length differences of these two type bonds are remarkable (about 0.15 Å) in Be and Mg doped systems, but the corresponding values in other cases are quite tiny (about 0.05 Å, similar to the case of undoped TiO_2_). This means the symmetry of local AEMO_6_ octahedron might evolve from *D*_4*h*_ to *O_h_* with the dopant atom from Be, Mg to Ca, Sr, and Ba, generating different crystal coordination fields. The data in Table 1 also indicates that the corresponding AEM-O bonds become longer with the increasing of the atomic number of the dopant AEM, which is consistent with the change of their ionic radiuses (see Table 1) [33]. Compared to the Ti-O bond lengths in undoped TiO_2_, the shrink of AEM-O bond lengths only can be found in the Be-doped system. From what have been illustrated above, we can see that a smaller dopant atom tends to pull the surrounding O atoms inward, whereas the larger dopant atom attempts to push the coordinated O atoms outward. This is similar to the previous studies for alkali and alkaline earth metal doped ZnO, as well as the Ni impurity in TiO_2_ [34,35].

**Table 1:** The bond lengths (Å) between alkaline earth metal atom and the six nearest neighbor O atoms in the doped anatase TiO_2_, and the averaged differences of the selected bond lengths between doped and pure anatase TiO_2_ (Δ, in Å). The ionic radiuses (in Å) of the alkaline earth metal elements are also listed.

Quantity	Ti	Be	Mg	Ca	Sr	Ba
AEM-O_1_	2.004	2.043	2.177	2.249	2.383	2.483
AEM-O_2_	2.004	2.042	2.178	2.248	2.383	2.483
AEM-O_3_	1.946	1.888	2.028	2.274	2.327	2.435
AEM-O_4_	1.946	1.888	2.028	2.276	2.328	2.434
AEM-O_5_	1.946	1.888	2.028	2.274	2.327	2.435
AEM-O_6_	1.946	1.888	2.028	2.276	2.328	2.434
Δ	0	–0.025	0.126	0.301	0.380	0.485
Ionic-radius	0.53	0.31	0.65	0.99	1.13	1.35

### 2.2. Formation Energy

The dopant formation energy is a widely used quantity to examine the relative stability of doped systems [36,37]. In this work, the formation energies were calculated by using the formula, *E*_form_ = *E*_doped_ – *E*_pure_ – μ_AEM_ + μ_Ti_, where the subscript AEM stands for the dopant atoms Be, Mg, Ca, Sr, and Ba, *E*_form_ is formation energy, *E*_pure_ and *E*_doped_ are the total energies of the undoped and AEM-doped anatase TiO_2_ model systems, respectively. μ_Ti_ and μ_AEM_ are the chemical potentials of Ti and AEM, respectively. In thermodynamic equilibrium with anatase phase, the chemical potentials of Ti and O must satisfy the relation μ(TiO_2_) = μ_Ti_ + 2μ_O_. However, it should be noted that the formation energy depends on growth conditions [38]. High (low) value of μ_Ti_ corresponds to Ti-rich (Ti-poor) conditions and also can be interpreted as O-poor (O-rich) conditions. Under O-rich conditions, the chemical potential μ_O_ is determined by the O_2_ molecule (μ_O_ = μ(O_2_)/2) and μ_Ti_ is calculated by the equilibrium relation μ_Ti_ = μ(TiO_2_) – 2μ_O_. Under Ti-rich growth conditions, μ_Ti_ is the energy of one Ti atom in bulk Ti (μ_Ti_ = μ_metal Ti_), and then μ_O_ can be obtained on the basis of the previous formula 2μ_O_ = μ(TiO_2_) – μ_Ti_. For Be, Mg, Ca, Sr, and Ba dopant impurities, the chemical potential μ_AEM_ is determined in terms of the relationship *n*μ_AEM_ = μ(AEM_*n*_O_*n*_) – *n*μ_O_ (*n* is the number of AEM and O atoms in the cell of AEM oxides). In terms of the calculated chemical potentials (see Table S1 in Supporting Information), the formation energies of AEM doped systems are given in Table 2. Both PBE and HSE06 results indicate that the doping under O-rich conditions is more energetically favorable than that under Ti-rich conditions. This is consistent with other previous works [39,40], meaning that substitution of lattice Ti atom in anatase TiO_2_ with a AEM atom is energetically favorable under O-rich growth conditions. It had been recognized that the smaller formation energy is, the more preferable to incorporate dopant to the host supercell is [41]. For the PBE results, the formation energy of Mg-doped TiO_2_ is the smallest among the doped systems under O-rich and Ti-rich conditions. This result can be interpreted by the comparable ionic radiuses of Mg and Ti as listed in Table 2. Also, it can be found that the formation energies increase with the increasing of ionic radius difference from Mg, generating the largest formation energy of Ba-doped system. In addition, the positive formation energies of the AEM doped anatase TiO_2_ indicate that the alkaline earth metal impurities are metastable states at local minimum. Whereas for the HSE06 results, the same conclusion and trend as those observed from the PBE results can be found, except that the smallest formation energy is the case of Ca. The dependence of formation energies on DFT functionals suggests that the formation energies of the systems under study are sensitive to the computational method of exchange term in total energy functional. Due to the better performance of HSE06 functional in the description of electronic structures, HSE06 functional might be more authentic than PBE functional for calculating formation energies.

**Table 2:** Calculated formation energies (eV) with PBE and HSE06 functionals, for Be, Mg, Ca, Sr, and Ba doped anatase TiO_2_.

Dopant Atom	GGA		HSE06	
	O-Rich	Ti-Rich	O-Rich	Ti-Rich
Be	3.50	8.74	5.89	11.66
Mg	1.80	7.04	3.38	9.15
Ca	2.20	7.44	1.75	7.52
Sr	2.76	8.00	2.31	8.08
Ba	3.68	8.92	2.74	8.51

### 2.3. Electronic Properties

#### 2.3.1. PBE Results

**Figure 2:**
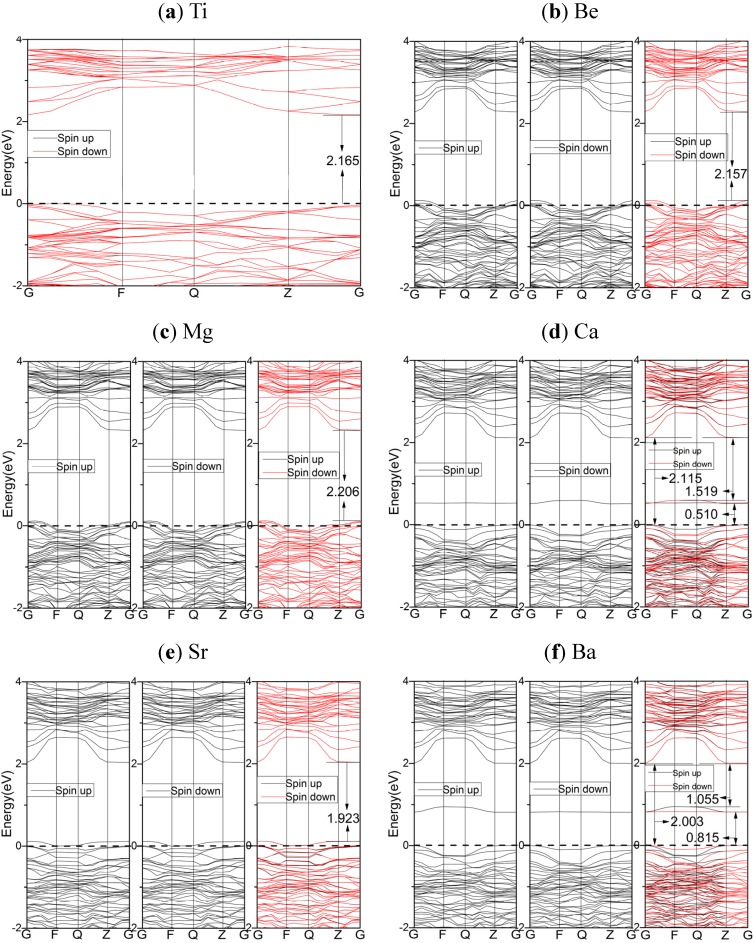
The spin polarized energy band structures of undoped and doped TiO_2_, calculated by using the PBE functional: (**a**) undoped TiO_2_; (**b**) Be-doped TiO_2_; (**c**) Mg-doped TiO_2_; (**d**) Ca-doped TiO_2_; (**e**) Sr-doped TiO_2_; (**f**) Ba-doped TiO_2_. The dashed lines indicate the Fermi energy.

Figure 2 gives the electronic energy bands of pure TiO_2_ and AEM-doped TiO_2_ calculated by using PBE.

Apparently, the spin-up and spin-down band structures are almost same for pure TiO_2_ and AEM-doped TiO_2_. This indicates the spin polarization effects can be ignored in these systems. In Figure 2, the calculated band gap of pure anatase TiO_2_ is about 2.17 eV, which is smaller than the experimental value (3.2 eV) due to the adopted GGA type functional. The calculated band gap of Be-doped TiO_2_ (2.16 eV) is about 0.24 eV smaller than that of undoped TiO_2_.

**Figure 3:**
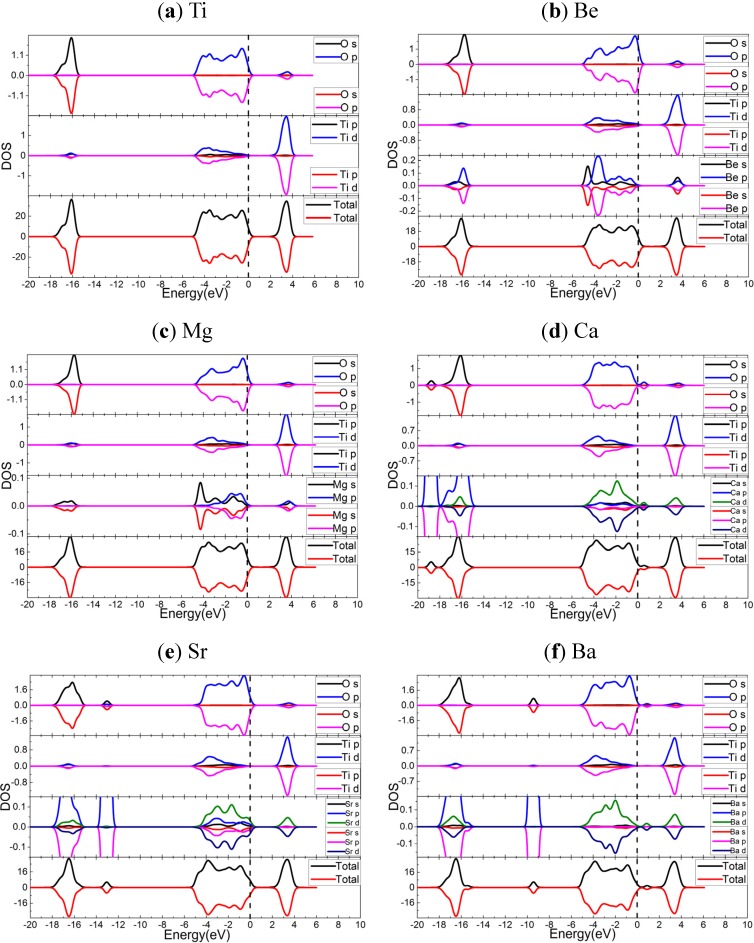
The spin polarized DOS and PDOS of doped and undoped TiO_2_, calculated by using the PBE functional: (**a**) undoped TiO_2_; (**b**) Be-doped TiO_2_; (**c**) Mg-doped TiO_2_; (**d**) Ca-doped TiO_2_; (**e**) Sr-doped TiO_2_; (**f**) Ba-doped TiO_2_. The dashed lines indicate the Fermi energy.

Apparently, Be and Sr dopants narrow band gaps without creating isolated states. This character is regarded to be more effective for photocatalytic activity [42,43]. More interestingly, the Ca and Ba doped TiO_2_ generate dopant states in band gap, which are about 0.51 and 0.82 eV above the Fermi level, respectively. Furthermore, in the cases of Ca and Ba doped systems, the slight difference of dopant states between spin-up and spin-down might mean that local spin polarization exists (see Figure S1). The Be, Mg, and Sr dopants move the Fermi level into VBs, and Ca and Ba dopant generate dopant states near the top of VBs.

In order to investigate the doping mechanism, the density of states (DOS) and partial DOS (PDOS) are presented in Figure 3. Again, the DOS and PDOS support that the spin polarization effects are quite tiny. For undoped TiO_2_, the VBs and the CBs are mainly composed of O 2*p*, Ti 3*d* orbitals, respectively. Also, the resonances of Ti and O orbitals in both VBs and CBs indicate the formation of Ti-O bonds. While for the doped cases, the *s* orbitals of AEM hybrid with *p* (for Be and Mg) or *pd* orbitals (for Ca, Sr, Ba) through the promotion of *s* electrons to *p* and *d* orbitals, generating coordination fields to interact with the nearest O. The electron densities and electron density differences (see Figures S2 and S3) can exhibit the variation of bonds and coordination fields. The orbital resonance between O and AEM suggests the AEM-O bonds formed. The VBs of doped systems contain the hybridized orbitals of AEM, and the CBs include the *sp* orbitals of Be and Mg, or the *d* orbitals of Ca, Sr, and Ba. The AEM dopant states in gap of Ca and Ba-doped cases might introduce small polaron [26]. In terms of deep energy level, the band-shifts in Be and Mg-doped cases are very tiny, but the Ca, Sr, and Ba dopants induce elevation of VBs, and generate dopant states in gap for Ca and Ba doped cases.

#### 2.3.2. HSE06 Results

**Figure 4:**
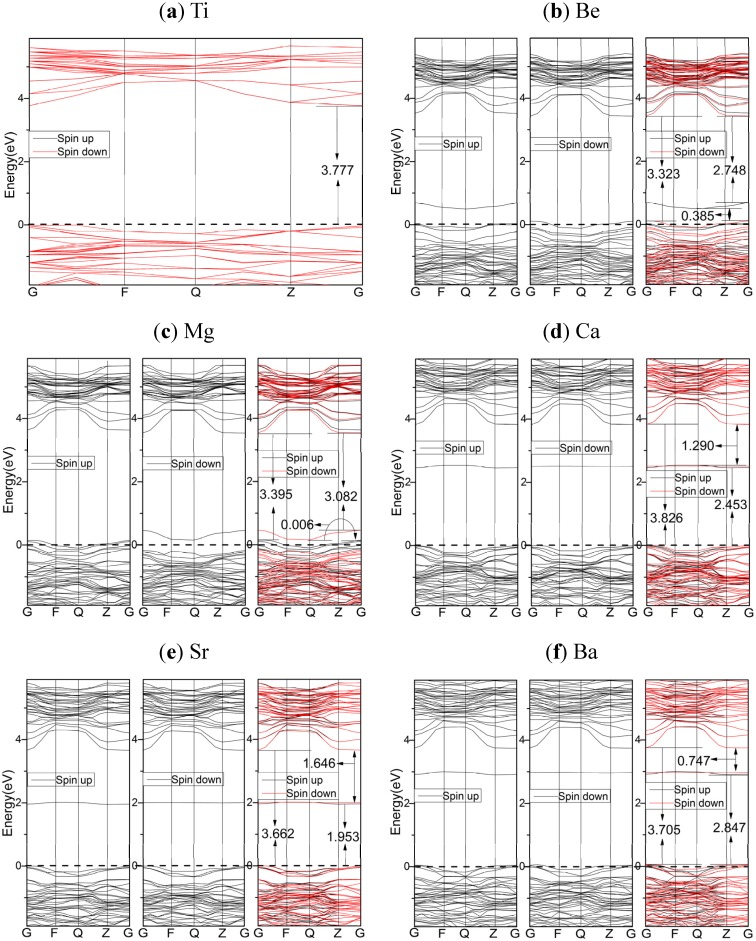
The spin polarized energy band structures of doped and undoped TiO_2_, calculated by using the HSE06 functional: (**a**) undoped TiO_2_; (**b**) Be-doped TiO_2_; (**c**) Mg-doped TiO_2_; (**d**) Ca-doped TiO_2_; (**e**) Sr-doped TiO_2_; (**f**) Ba-doped TiO_2_. The dashed lines indicate the Fermi energy.

The electronic band structures of HSE06 results for pure TiO_2_ and AEM-doped TiO_2_ are presented in Figure 4. The band structures of HSE06 results for TiO_2_ are quite similar to that of PBE results, except the gap values. However, for doped systems, the dopant states are induced in intrinsic band gap, and they are above the top of VBs about 0.385, 0.006, 2.453, 1.953, and 2.847 eV for Be, Mg, Ca, Sr, and Ba, respectively. Dopants and defects in TiO_2_ usually induce dopant states in the gap [26,32]. Because the PBE results missed the dopant states for the cases of Be, Mg, and Sr, the HSE06 functional is more reliable than PBE functional due to the generation of dopant states. In the cases of Ca, Sr, and Ba, the significant differences between dopant state energy and Fermi level suggest the dopant species induce the polaron, which can remarkably change the electron density (see Figures S2 and S3). Furthermore, for the cases of Ca, Sr, and Ba-doped systems, the quite similar band structures between spin-up and spin-down mean that spin polarization effects can be ignored. Whereas for the cases of Be and Mg-doped systems, the dopant states are spin up and spin down for Be and Mg, respectively. The broken of spin symmetry indicates the existence of spin plolarization which could be supported by spin densities presented in Figure S1. It was also found for the case of Mg by LDA + U calculations [27].

**Figure 5:**
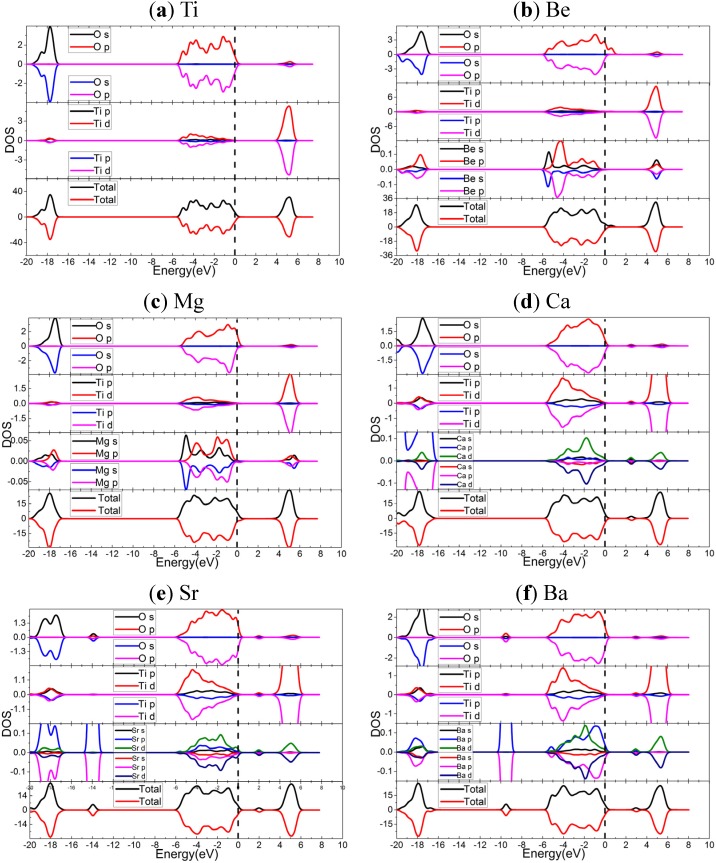
The spin polarized DOS and PDOS of both doped and undoped TiO_2_, calculated by the using HSE06 functional: (**a**) undoped TiO_2_; (**b**) Be-doped TiO_2_; (**c**) Mg-doped TiO_2_; (**d**) Ca-doped TiO_2_; (**e**) Sr-doped TiO_2_; (**f**) Ba-doped TiO_2_. The dashed lines indicate the Fermi energy.

Figure 5 shows the DOS and PDOS of HSE06 results. For undoped TiO_2_, the components of CBs and VBs, as well as the orbital resonance are very similar to that of PBE results. For the doped cases, the components of CBs and VBs, orbital hybridization of dopant atom, and orbital resonance are also similar to those of PBE results. From the above mentioned points view, the PBE and HSE06 functionals generate quantitatively different results. However, the most important difference between PBE and HSE06 results is dopant states. The PDOS of Be and Mg doped systems indicate that the dopant states in these systems are resulted from the local spin polarization of O near Fermi energy. But for the cases of Ca, Sr, and Ba, the dopant states include the contribution from the *p* orbials of O, *d* orbitals of Ti, and hybridized orbitals of AEM, suggesting the delocalized character of dopant states. The components of VBs and dopant states mean the AEM dopants are active in inter-band transitions induced by lower energy excitations. In terms of deep energy level, Be and Mg-doped anatase TiO_2_ shift the VB to higher energy region, and thus reduce band gap. For the cases of Ca, Sr, and Ba, the VBs also shift to higher energy, and the energies of dopant states in gap are quite higher than Fermi levels.

It was pointed out that the dopant-induced supplementary charges incorporated in the anatase lattice by Mg dopant could be compensated by the generation of Ti vacancies [22], which is similar to the case of Nb-doped TiO_2_ [44]. The electron densities (Figure S2) and electron density differences (Figure S3) have significant difference between anatase TiO_2_ and AEM doped anatase TiO_2_. Therefore, combined with the analysis of energy bands and DOS/PDOS, the AEM dopants might introduce a polaron [26].

### 2.4. Optical Properties

Optical properties are very important to develop novel optic-electronic materials. The optical properties can be characterized by complex dielectric function ε(ω). The absorption coefficient α(ω) can be obtained from the real part ε_1_ (ω) and imaginary part ε_2_ (ω) of ε(ω) [27,45],

α(ω)=2ω[ε12(ω)+ε22(ω)−ε1(ω)]12

The ε_2_ (ω) can be calculated from the momentum matrix elements between the occupied and unoccupied states of wavefunctions,

$$\varepsilon_{2}\left(\omega \right)=\left(\frac{4\pi^{2}e^{2}}{m^{2}\omega^{2}}\right){\sum_{i,j}\int \langle i\left|M\right|j\rangle }^{2}f_{i}\left(1-f_{j}\right)\delta \left(E_{f}-E_{i}-\omega \right)d^{3}k,$$

where M is the dipole matrix, *i* and *j* are the initial and final states respectively, *f_i_* is the Fermi distribution function for the *i*th state and *E_i_* is the energy of electron in the *i*th state. The ε_1_ (ω) can be evaluated from ε_2_ (ω) by using the Kramers-Kroning transformation in the form,

$$\varepsilon_{1}\left(\omega \right)=1+\frac{2}{\pi }P\int_{0}^{\infty }\frac{\omega^{\prime }\varepsilon_{2}\left(\omega^{\prime }\right)d\omega^{\prime }}{\left({\omega^{\prime }}^{2}-\omega^{2}\right)},$$

where *P* is the principal value of the integral.

**Figure 6:**
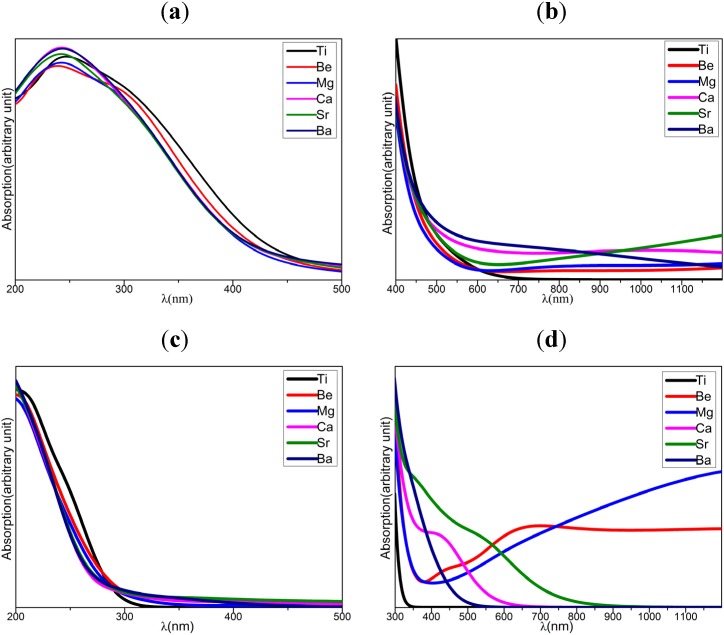
The optical absorption spectra of both doped and undoped TiO_2_, calculated by using PBE (**a**,**b**) and HSE06 (**c**,**d**) functionals.

The optical absorption spectra calculated by using PBE (*a* and *b* in Figure 6) and HSE06 (*c* and *d* in Figure 6) for the undoped and AEM-doped TiO_2_ systems are shown in Figure 6. Experimentally, the optical band gap is determined by the excitation energy intercept at absorption edges in absorption spectra, and the optical band gaps exhibited about 3.20 and 3.27 eV for the TiO_2_ and Mg-doped TiO_2_ [22]. In terms of the excitation energy intercept which is obtained by tangent line at absorption edges along absorption curve from *a* and *c* in Figure 6, the wavelengths, corresponding to excitation energy intercept for Mg-doped TiO_2_, are shorter than those of undoped TiO_2_. This means the calculated optical band gap of Mg-doped TiO_2_ is larger than that of undoped TiO_2_, agreeing with the experimental results [22].

Through the tangent line at absorption edges along absorption curve (*a* and *c* in Figure 6), both PBE and HSE06 results generate the wavelengths at excitation energy intercept for AEM-doped TiO_2_ are shorter than that of undoped TiO_2_. This suggests that the AEM dopants in TiO_2_ increase the optical band gaps. Apparently, the AEM dopant effects in optical band gap are quite different from those in electronic band gaps. It is reasonable that the electronic band gap is based upon single particle approximation, while the optical absorption involves the excitation beyond single particle picture. On the other hand, the difference also might result from that some AEM-doped TiO_2_, such as Mg-doped system [22], is an indirect transition band-gap semiconductor. Furthermore, the PBE results (*b* in Figure 6) indicate that the enhancement of absorbance in visible and near IR region by Ca, Sr, and Ba dopants are more significant than that of Be and Mg dopants. Meanwhile, the HSE06 results (*d* in Figure 6) show that, the Ca, Sr, and Ba dopants are superior to Be and Mg dopants to enhance absorbance in visible region, inversely, the Be and Mg dopants are better than Ca, Sr and Ba dopants for the improvement of absorbance in near-IR region. This agrees with the LDA + U calculation for the case of Mg [27]. The absorbance enhancement in visible and near-IR region results from the dopant states in gap. So, the Be/Mg and Ca/Sr/Ba dopants can compensate optical absorbance in different wavelength region. The different performances of Be/Mg and Ca/Sr/Ba in visible and near-IR region present that the co-doped system may possess prominent optical absorption properties.

## 3. Computational Methods

**Figure 7:**
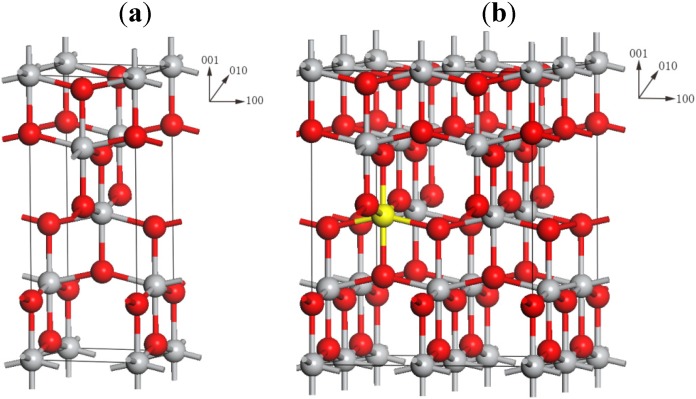
The crystal structure of computational model: (**a**) the conventional cell of anatase TiO_2_; (**b**) the 48-atom 2 × 2 × 1 supercell. Ti atoms are represented by light gray circles and O atoms are represented by red circles. The yellow circle represents the Ti atom which is chose to be substituted by an AEM atom.

The DFT calculations in this work were carried out using the CASTEP package [46]. The GGA type functional PBE [47], combined with ultrasoft pseudopotentials (USPP) [48], was applied to determine lattice parameters. In this procedure, the electronic wave function was expanded in the plane wave basis sets with the kinetic energy cutoff of 500 eV. The full optimization of the conventional cell of the pure anatase TiO_2_ (see Figure 7a) was performed to verify the accuracy of computational method. The calculated lattice parameters are *a* = *b* = 3.796 Å, and *c* = 9.722 Å, which agree well with other DFT calculations [40,49,50] and the experimental values [51,52]: *a* = *b* = 3.785 Å and *c* = 9.514 Å, indicating that our calculation parameters are appropriate.

Since the X-ray diffraction data demonstrated that Mg occupied the lattice Ti-site in Mg-doped anatase TiO_2_ [22,23], the model systems of AEM-doped anatase TiO_2_ were constructed by using the substitution of lattice Ti with an AEM (AEM = Be, Mg, Ca, Sr, and Ba) atom on the basis of the 48-atom 2 × 2 × 1 anatase supercell (see Figure 7b) [53], corresponding 6.25% doping level. For the relaxation calculations of the supercell models by using PBE and USPP, a 3 × 3 × 3 k-mesh [54] was employed and the convergence threshold for self-consistent iteration was set at 1 × 10^−6^ eV/atom.

To choose appropriate functional, we calculated the band gap of pure anatase TiO_2_ with the experimental lattice parameters by using four kinds of hybrid functionals: PBE0 [55], B3LYP [56], HSE03 [29], and HSE06 [30]. The hybrid functional calculations were performed with norm-conserved pseudopotential (NCPP) with the kinetic cutoff energy of 750 eV. The calculated band gaps (*E*_g_) are summarized in Table 3. The PBE0 and B3LYP results indicate that the Hartree-Fock contents in exchange (25% in PBE0 and 20% in B3LYP) have significant effects on *E*_g_, and the screened range separated hybrid functional HSE06 generates the *E*_g_ about 3.69 eV, which agrees with the previous HSE06 result [57], and it is the closest result to the experimental value [58]. Therefore, we choose the HSE06 functional to calculate the spin polarized electronic structures and related properties of undoped and AEM-doped anatase TiO_2_ based upon the geometries optimized with the PBE functional. Meanwhile, the calculations by PBE functional were also performed in order to disclose the functional effects on electronic structures and related properties.

**Table 3:** Calculated band gaps (*E*_g_, eV) for pure anatase TiO_2_ by using different hybrid functionals.

Functional	PBE0	B3LYP	HSE03	HSE06	Experiment
Band gap (eV)	4.46	4.00	3.71	3.69	3.20

## 4. Conclusions

In this work, the dopant formation energies, electronic structures, and optical properties for AEM doped anatase TiO_2_ are investigated by using DFT calculations, performed with the screened hybrid functional HSE06 and generalized gradient approximation functional PBE. Our results indicate that

- Viewing from methodology, by combining the previous studies with our results, including band gaps and the dopant states, HSE06 provides a better description of the electronic structures.
- The formation energies indicate that the substitution of a lattice Ti atom with an AEM atom is more energetically favorable under O-rich growth conditions than those of Ti-rich cases.
- Based upon the HSE06 results, the analysis of electronic structures suggest that, AEM dopants shift the VBs to higher energy, and the more important is that the energies of dopant states for the cases of Ca, Sr, and Ba are quite higher than the top of VBs, while the Be and Mg dopants result into the spin polarized dopant states near Fermi levels. The components of VBs, CBs and dopant states support that the AEM dopants are active in inter-band transitions induced by lower energy excitations, which is important for the application of solar energy.
- Compared with anatase TiO_2_, the AEM dopants shift the absorption to longer wavelength and improve optical absorbance in visible and near-IR region. The Ca, Sr, and Ba dopants are superior to Be and Mg dopants to enhance absorbance in visible region, inversely, the Be and Mg dopants are better than Ca, Sr and Ba dopants for the improvement of absorbance in near-IR region. The compensating optical absorbance of Be/Mg and Ca/Sr/Ba dopants in different wavelength region present that the Be/Mg and Ca/Sr/Ba co-doped anatase TiO_2_ may possess prominent optical absorption properties.

Extensions of the present work would be to explore the local spin polarization and polaron effects of AEM doped anatase TiO_2_. A challenge is that, the larger super cell and more dopant atoms should be required, the spin configurations also should be considered, which will be computationally demanding. This will be systematically investigated in the future work.

## Supplementary Materials

Supplementary materials can be accessed at: http://www.mdpi.com/1996-1944/8/8/5508/s1.
