# Supplementary material for: The Electronic Structures and Optical Properties of Alkaline-Earth Metals Doped Anatase TiO2: A Comparative Study of Screened Hybrid Functional and Generalized Gradient Approximation
Source: Materials (Basel). 2015 Aug 24;8(8):5508–25. doi: 10.3390/ma8085257 (PMC5455518; doi:10.3390/ma8085257)
Supplement: Supplementary file 1 [file materials-08-05257-s001.pdf]

Supporting Information

Table S1. The calculated chemical potential values (in eV) of Ti, O, and alkaline-earth metals (AEM) for AEM-doped anataseTiO_2.The larger difference of chemical potential for Mg results from the different valence electron configurations for pseudopotential.

Element	GGA		HSE06	
	O-rich	Ti-rich	O-rich	Ti-rich
O	–434.006	–439.243	–427.883	–433.623
Ti	–1613.601	–1603.126	–1583.821	–1572.341
Be	–36.742	–31.505	–38.155	–32.416
Mg	–979.835	–974.598	–1602.834	–1597.095
Ca	–1008.095	–1002.858	–1004.297	–998.557
Sr	–842.413	–837.176	–844.573	–838.833
Ba	–704.929	–699.692	–699.695	–693.955

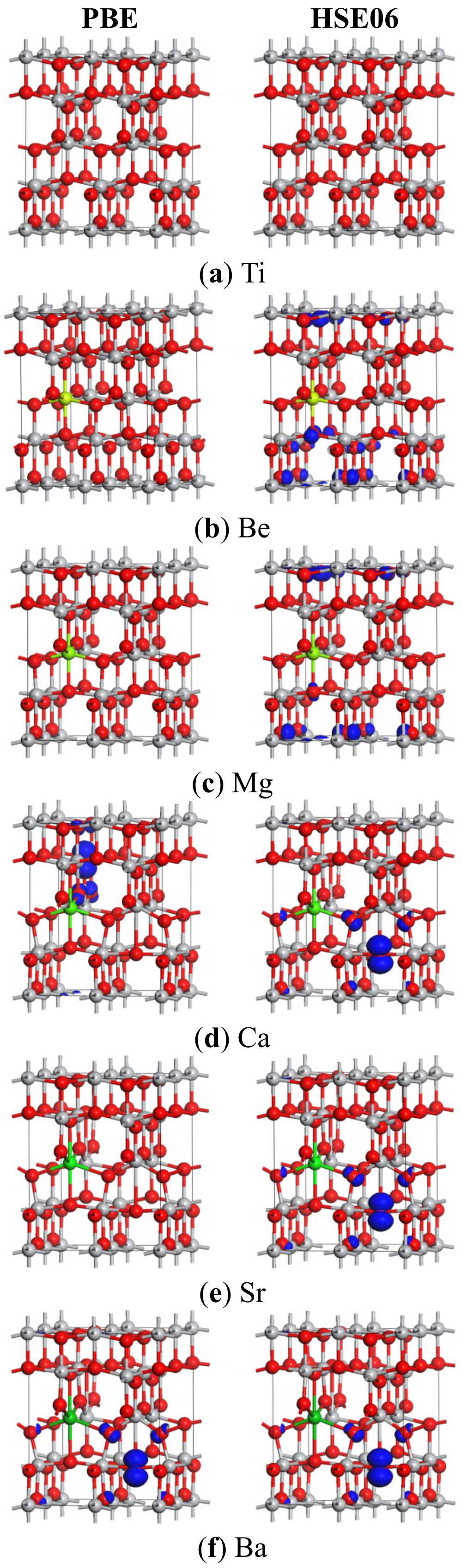

Figure S1. The spin density differences of undoped and doped TiO_2, calculated by using the PBE and HSE06 functionals, and the dopant atom was highlighted (isodensity contour = 0.05 a.u.): (**a**) undopedTiO_2; (**b**) Be-doped TiO_2; (**c**) Mg-doped TiO_2; (**d**) Ca-doped TiO_2; (**e**) Sr-doped TiO_2; (**f**) Ba-doped TiO_2.

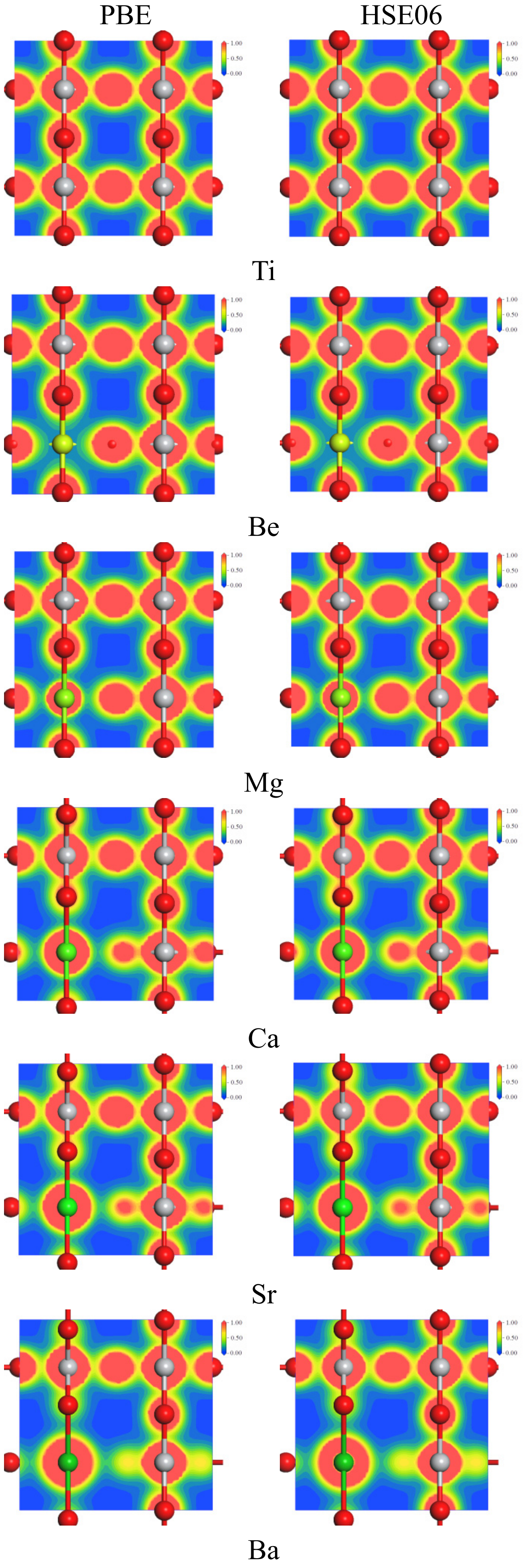

Figure S2. The electron densities of doped and undopedTiO_2 cut along 001 surface through dopant atom, and the dopant atom was highlighted.

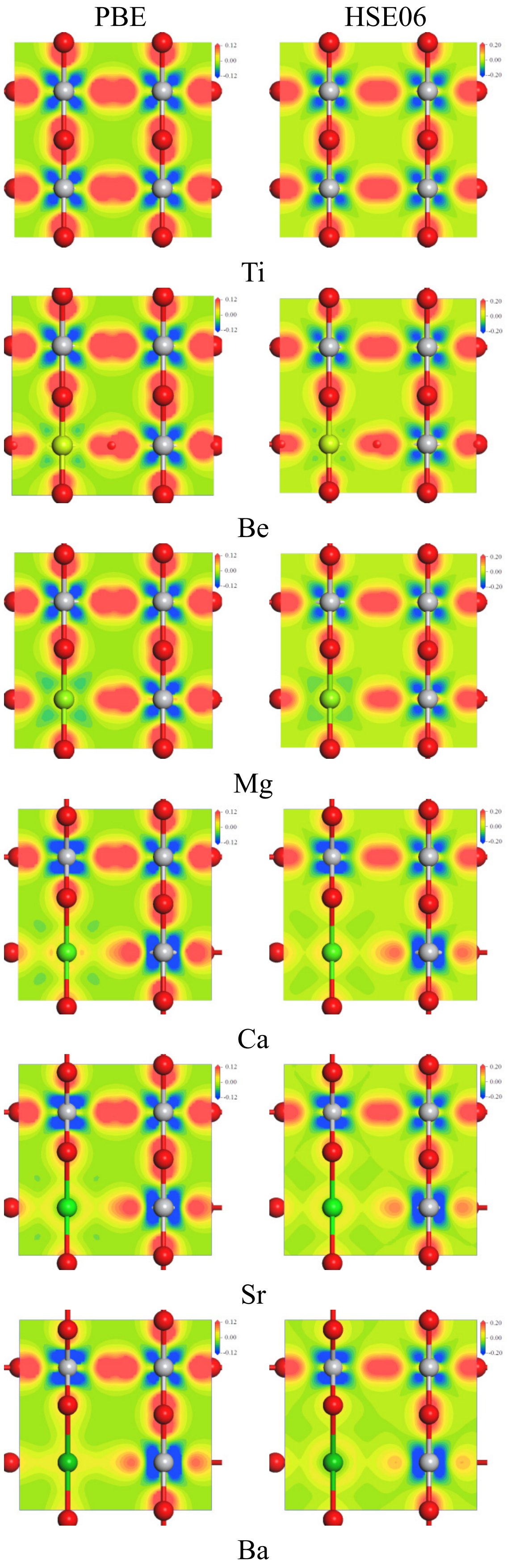

Figure S3. The electron density differences of doped and undoped TiO_2 cut along 001 surface through dopant atom, and the dopant atom was highlighted.
